# Appraisal of Low-Cost Pushbroom Hyper-Spectral Sensor Systems for Material Classification in Reflectance

**DOI:** 10.3390/s21134398

**Published:** 2021-06-27

**Authors:** Steven Hobbs, Andrew Lambert, Michael J. Ryan, David J. Paull, John Haythorpe

**Affiliations:** 1 School of Engineering and Information Technology (SEIT), University of New South Wales Canberra, Northcott Drive, Canberra, ACT 2600, Australia; a-lambert@adfa.edu.au; 2 Capability Associates Pty Ltd., Canberra, ACT 2600, Australia; mike.ryan@ieee.org; 3 School of Science, University of New South Wales Canberra, Northcott Drive, Canberra, ACT 2600, Australia; d.paull@adfa.edu.au; 4 Mars Society Australis, Clifton Hill, VIC 3068, Australia; john.haythorpe@gmail.com

**Keywords:** optics, spectrometry, cubesat, STEM, pushbroom, Bayer filter

## Abstract

Near infrared (NIR) remote sensing has applications in vegetation analysis as well as geological investigations. For extra-terrestrial applications, this is particularly relevant to Moon, Mars and asteroid exploration, where minerals exhibiting spectral phenomenology between 600 and 800 nm have been identified. Recent progress in the availability of processors and sensors has created the possibility of development of low-cost instruments able to return useful scientific results. In this work, two Raspberry Pi camera types and a panchromatic astronomy camera were trialed within a pushbroom sensor to determine their utility in measuring and processing the spectrum in reflectance. Algorithmic classification of all 15 test materials exhibiting spectral phenomenology between 600 and 800 nm was easily performed. Calibration against a spectrometer considers the effects of the sensor, inherent image processing pipeline and compression. It was found that even the color Raspberry Pi cameras that are popular with STEM applications were able to record and distinguish between most minerals and, contrary to expectations, exploited the infra-red secondary transmissions in the Bayer filter to gain a wider spectral range. Such a camera without a Bayer filter can markedly improve spectral sensitivity but may not be necessary.

## 1. Introduction

Optical spectrometers have been invaluable instruments for understanding the physical properties of materials for the past two centuries [1]. Historically, many Earth-based and planetary spacecraft have included multispectral sensors that were able to collect data in a limited set of spectral bands [2]. Although able to return information across parts of the spectrum in broad channels, these bands are limited such that subtle absorption characteristics are often missed, as this information is merged with other data within the channels to which the sensor is sensitive. This in turn limits the effectiveness of these instruments in identifying and characterizing mineralogy.

Hyperspectral sensors overcome this limitation by producing images in which each pixel contains the spectral signature of a scene in hundreds of narrow spectral bands [3]. Panchromatic and RGB images are restricted to one and three channels, respectively, however hyperspectral images are captured in many channels. Hyperspectral sensors typically capture these spectral bands as a series of monochromatic images, which are then stacked to form a hyperspectral cube of spatial and spectral dimensions. Prior to detection, a necessary component of this process is to divide incoming radiation into narrow sections, representing bands a few nanometers apart. This process, while increasing spectral sensitivity, vastly reduces the number of photons at each pixel, requiring increased sensor sensitivity or exposure time.

Recent advances in hyperspectral technology have empowered the development of relatively small, scientifically useful instruments for the diverse range of applications for stakeholders with limited budgets. Applications of these sensors have included agricultural vegetation modelling [4,5]; algal bloom investigation [6] and even deep space applications [7] as well as the possibility of carrying out remote geological surveys [8]. Multiple technologies have been devised and built to support these applications, including liquid crystal tunable filters [9], tunable Fabry–Perot filters [10] and diffractive optics [11,12]. Diffractive pushbroom hyperspectral sensors have found utility in low-cost research applications [11,12] and do not require electronically driven filters.

The ready availability of commercial-off-the-shelf complementary metal oxide semiconductor (CMOS) and charge coupled device (CCD) sensors has recently opened new avenues for low-cost hyperspectral sensors. Science, technology, engineering and mathematics (STEM) and citizen science designs have been trialed and used in field settings [13,14]. Despite such applications, performance comparisons with commercial-grade spectrometers are missing from the literature. These analyses are an important consideration in identifying the utility of sensor/optics combinations for remote sensing particularly, as is of interest to this work, in the field of planetary research in extraterrestrial-analogous environments.

This paper explores the application of hyperspectral technology to the appraisal of spectral responses of minerals of interest gathered during surface-based planetary robotic lander missions [15,16,17]. An attribute of lander-based spectral sensing is that the proximity of the sensor to the object of interest minimizes attenuation due to atmospheric effects as radiative or reflective energy reduces with increasing distance from the target. Furthermore, orbital sensors [18] suffer short integration times and higher frame rates that compensate for travel velocity, which is not so much a problem for localized systems. The disadvantage is the sensor must be placed in close proximity to the target of interest to be effective. However, lander missions have used space-based remote sensing to assist with targeting scientifically interesting sites [15,16]. Testing of lander-based hyperspectral sensors in extraterrestrial-analogous environments on Earth allows for development of low-cost sensors where sensitivity and compensation for atmospheric losses are less of a consideration for sensor design.

In this work, the ground-vehicle paradigm is used to trial custom-built hyperspectral pushbroom sensors in the field. The spectral ranges of sensors were constructed in order to cover the 600–800 nm portion of the electromagnetic spectrum which provides the ability to detect the phenomenology of minerals found on the Moon, Mars and asteroids [7,19,20,21,22]. Three sensor options were evaluated to determine their ability to detect these materials and cost. Additionally, the application of extraterrestrial-analogous testing reduces space qualification engineering costs, enabling the resulting instrument designs to be within the budget of science, technology, engineering and mathematics (STEM) outreach activities or small organizations such as The Mars Society. We describe this method, the chosen sensors, system design, and show the requisite performance in ground tests alongside scientific grade spectrometers.

## 2. Methods

### 2.1. Sensor Design

A number of designs were considered for the spectral instrument. Fabry–Perot Interferometers (FPIs), which have found application in hyperspectral imaging, can use two opposing reflective surfaces separated by an air gap or liquid crystal filters [9,10] to discriminate between wavelengths. This requires the capture of large numbers of bands across the spectral dimension, with images separated in time [9] if liquid crystal filters are used.

Whiskbroom (single pixel) sensors use a mirror or gimbal mounted detector moving back and forth across a scene to capture a one-dimensional row of information. Additional movement, either through the orbit of a satellite if the whiskbroom is mounted in orbit, or a secondary motor, enable a two-dimensional image to be built of a scene of interest [23,24]. While the requirement for a single sensor may seem attractive from processing electronics overhead and dynamic range perspective, the necessary additional mechanical parts, such as the secondary motor, increase the risk of hardware failure and limit speed.

Pushbroom sensors use a two-dimensional array of detector elements, such as a CMOS image sensor, to capture spatial and spectral information. Physical motion of the sensor, in addition to a slit and dispersing optics such as a diffraction grating or prism, generates the second spatial axis in these sensors. The slit generates a one-dimensional image of the scene, with component wavelengths spread across to the additional dimension. Physical motion of the sensor through a pan motor, motion of a host platform such as a drone, or orbit velocity of a satellite, reconstitutes the image. The resulting 2D image, combined with a third dimension created by stacking images, contains the spectral composition of each pixel in the image.

The limited availability and high price of ready-made hyperspectral sensors precluded their consideration for this work. Tunable liquid crystal or FPI filters were considered, however these were deemed to be too complex for ready manufacture in a STEM context. Previous STEM and open-source research have favored LED-based multispectral; or diffractive-based spectrometer designs [11,12,25,26], which have taken advantage of readily accessible optical components and may be built using modest manufacturing equipment such as 3D printers, and are sufficiently robust for use in field environments [12]. Additionally, the design of the sensors only requires electronics to support the imaging sensor, but still requires smooth, continuous movement in order to generate a hyperspectral cube, and therefore requires sequential image capture from the image sensor to build the scene [18]. The optics must account for the amount of incident radiation and deliver it to the slit in an efficient coupling.

Low-cost versions of this sensor have been designed for public outreach, placing the science of spectroscopy into the hands of everyday people and promoting interest in science [27]. Additionally, pushbroom sensors have been designed for drone and cubesat research [11].

### 2.2. Sensor Considerations

The end purpose of the hyperspectral sensor in this work is to enable low-budget groups, such as schools or research societies, to conduct meaningful terrestrial analogue remote sensing and contribute findings to the scientific literature, while not necessarily expecting such sensors to perform well in satellites and space vehicles. In order to constrain hyperspectral imager design from a range of potential solutions, nine constraints were devised. This restricted suitable designs in the context of developing a low-cost hyperspectral instrument suitable for carriage on a small-scale vehicle with limited size, weight and power constraints, and applicable for a space analogue environment [28]. Table 1 lists the requirements for this project directed towards low-cost, small-size, low-weight and low-power operation.

The choice of the imaging sensor is critical to best satisfy the constraints, while also being of low cost and able to empower the analogue testing described above [29]. In addition to possessing a sensitivity of the 600–800 nm wavelength range required for edge of red analysis, on-specialist assembly and the ability to be controlled by a single board computer was required (Table 1).

Three COTS sensors met the requirements (satisfying the criteria above), and related to open-source scientific applications in previous research [11,12,30]. These include the 5-megapixel Raspberry Pi v1 camera (Omnivision OV5647 sensor, raspberrypi.org, UK), the 8-megapixel Raspberry Pi v2 camera (Sony IMX 219 sensor, raspberrypi.org, UK) and a ZWO Mini Astrocam (On AR0130 sensor, ZWO, China) [31].

The Raspberry Pi (RPICAM) version 1 and 2 cameras use Bayer filters for color sensing at differing resolutions and frame rates and have been extremely popular and easy to use with science enthusiasts [26,32]. An important consideration of the utility of a particular sensor for a hyperspectral instrument is the presence of a Bayer filter which exists on most color RGB sensors [33]. Color cameras possessing Bayer filters are very common and low-cost, but these can also degrade the sensor’s spectral response through absorption of incident radiation. This is particularly apparent when the filter channels cross over (blue/green, green/red) in which case up to 50% of the incident radiation is absorbed and which limits the detectability of the full spectrum [34,35]. Monochrome sensors do not possess a Bayer filter, and their spectral response is driven predominantly by the sensitivity within the silicon substrate of the sensor [36]. While monochrome sensors are definitely advantageous in this regard, they are not as mass produced as their color counterparts. This availability must be factored into measures of utility. The ZWO Mini Astrocam represents an affordable monochrome camera used for amateur astronomy applications.

### 2.3. Noise Considerations

The ability of a sensor to distinguish spectra phenomena is also influenced by additional limitations of the sensor itself. Noise equates to undesired pixel values generated with a received signal affect CCD and CMOS sensors. This can originate from thermal effects, where an increase in temperature increases the amount of random noise generated by the sensor [37,38]. Thermal noise can particularly degrade the sensor signal during long exposures and long readout times, and methods to manage sensor temperatures have been used in previous cubesat missions [39]. In the case of this application, longer exposure times and low signal from the dispersive sensor design would be a primary consideration. Shot noise results from statistical variations in photon arrival rate in striking the sensor and influences sensor signal in high photon counts [40]. While fast readout rates contribute readout noise and hence restrict the usable frame rate, this effect is less apparent in CMOS sensors due to the on-site digitization of the signal.

The strength of the incident signal must exceed the noise floor of the sensor in order for it to be registered as a meaningful result. This is particularly of interest to spectral sensors, where large numbers of images are produced with low incident radiation levels [41,42].

Noise analysis has previously been undertaken on the Raspberry Pi v2 camera [32]. Dark frame imagery assessment at 25 °C found that DC pedestal offset associated with dark current combined with noise increased with an increase in ISO settings from 100 to 600 (maximum digital number, DN, 121.8 to 442.4, 5 ms exposure; maximum DN 417.74 to 1021.43, 50 ms exposure). These values identified in that work were considered appropriate for producing scientifically useful results. It should be noted that the IMX219 chip of the Raspberry Pi v2 camera includes non-light sensitive pixels that can alter the black level such that a raw 10-bit image taken in zero light conditions will default to a digital number (DN) of 64 (0–63 contain no data in this instance). This result explains the minimum DN values obtained by previous research [32,41] and can be altered utilizing the register set of the sensor in software.

### 2.4. Calibration

Calibration is a critical component of spectroscopy in order to obtain meaningful reflectance results while also accounting for variations in incident light and sensor characteristics. Two calibration methods were performed on the pushbroom sensor: wavelength and radiometric. Wavelength calibration entailed ensuring captured spectra corresponded with the appropriate wavelengths of incident light. The use of compact fluorescent lamps (CFL) has been popular for wavelength calibration by researchers [11,43]. This is due to their ready availability, low cost and high spectral response covering multiple wavelengths. Light from a CFL was reflected onto a specular light reflector in order to obtain images of spectral peaks [43]. These spectral peaks, occurring at specific wavelengths, were then used to calibrate the sensor.

Radiometric calibration was conducted by using a 100 mm square Teflon sheet as a white specular reflector and operating the sensors under direct sunlight. To ensure lighting was consistent, cloud-free days were chosen for the trial, with timing coinciding with mid-morning to minimize atmospheric absorption. Previous research has used the following formula to extract reflectance spectra (R_f_) from sensor readings [44]:

R_f_ = (R_s_ − S_d_)/(R_r_ − S_d_) (1)

where R_s_ is the reflected energy from the target, S_d_ is the DC systematic pedestal signal associated with dark frames, and R_r_ is the reflected energy from a specular white target. Successful calibration required that the specular white target was subject to the same lighting conditions as the materials to be identified, and as such was imaged in the same scene as the other material samples. Dark frames were used for S_d_ and were obtained by capturing an exposure in the absence of light with the camera sensor prior to spectral collection in a similar manner of that performed in previous research [32,41,43]. For consistency and to manage thermal noise effects, sensor temperatures were kept at 20 °C.

### 2.5. Sample Capture

Consideration was given as to the number and types of materials used to characterize the sensors for VIS/NIR utility, particularly in the 600–800 nm (hereafter termed “ferric response”) portion of the spectrum. Color swatches of a known spectral value have been used in previous research to assist in measuring spectral output from sensors, including lander missions flown to Mars since the 1970s [15,17,22], multispectral [26] and hyperspectral applications [30]. The utility of three cameras for pushbroom sensor designs was trialed with minerals exhibiting phenomenology in the ferric response portion of the spectrum and that have been found in the inner solar system [15,16,45]. Previous research into these minerals has provided a critical understanding of the inner Solar System, particularly Mars and has shaped the design of missions for decades [15,17,22]. Additional materials included white and grey specular calibration targets, a custom color calibration chart, and vegetation samples.

**Figure 1:**
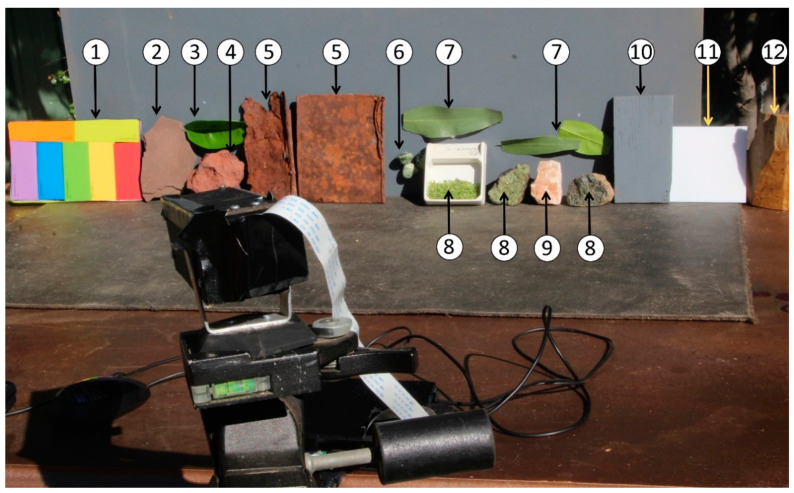
Outdoor target and calibration setup. Numbered materials are explained in the text.

Figure 1 shows an overview of the target capture for the custom pushbroom sensor. The sensor itself was mounted on a camera tripod and panned clockwise to capture the scene. The following materials (as shown in Figure 1) were used in this activity to demonstrate the appraisal:

(1) a custom-made color chart used to assist in sensor calibration in a similar method to that used in previous hyperspectral research and carried aboard Mars landers [15,17,22,30];
(2) iron oxide-rich mudstone for further characterization of spectral properties of these mineral types [16,17,19];
(3) non-deciduous citrus lemon leaf (labelled Lemon Leaf) used to assist in appraising sensor performance for potential terrestrial vegetation applications [30,46];
(4) iron oxide-rich hematite, analogous to surface minerals found on Mars and of particular interest to remote sensing research of the surface of this planet [15,16,17,21,22];
(5) rust samples, used to further characterize the sensor’s performance for identifying iron-oxide rich materials and compare with the mudstone and hematite samples [16,17,19];
(6) green serpentine samples, analogous to extraterrestrial minerals as suggested in studies of Mars minerals [47,48]; and
(7) non-deciduous Eucalyptus sp. leaf samples (labelled Eucalyptus Leaf), to compare with the Lemon Leaf sample for vegetation utility appraisal [30,46].

Additional analogous minerals used in this research included the following:

(8) three different types of olivine analogous to those identified elsewhere in the Solar System and of interest to understanding off-Earth geology [49,50]: olivine pebbles (referred to as Olivine 1) and two solid samples (referred to as Olivine 2 and Olivine 3, respectively); and
(9) gypsum, also relevant to extraterrestrial mineralogy [51].

Materials used to assist with calibration included the following:

(10) grey; and
(11) white specular reflectors. The spectra from these reflectors were analyzed using a laboratory grade Spectral Evolution SR-3500 spectroradiometer to confirm that they exhibited consistent, flat spectra.
(12) A goethite-rich sample (12), hereafter referred to as Goethite, completed the analyzed samples.

**Figure 2:**
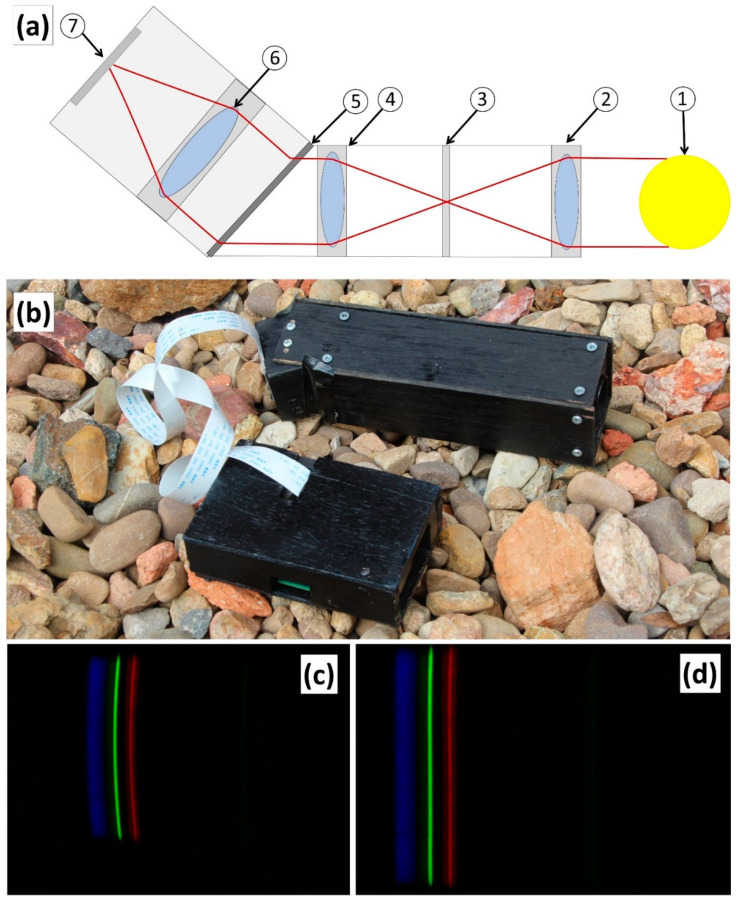
(**a**) RPi pushbroom sensor design showing (1) sample source (2) objective lens (3) slit (4) diffraction grating (5) collimating lens (6) camera lens (7) camera sensor. The optical pathway is shown in red. (**b**) Completed RPi pushbroom sensor with control unit. (**c**) Raw CFL result displaying smile artifact. (**d**) Corrected output.

Figure 2a shows the chosen design of the pushbroom sensor. Incident light from the source (1) is focused by a 30 mm diameter objective lens with a 60 mm focal length (2). This light passes through a 150 um width slit (3) before entering a C-mount-sized collimating lens (4). A 600 line/mm transmission diffraction grating (5) passes the light through the camera optical system (6) before being captured by the sensor (7). Figure 2b shows the completed pushbroom sensor with Raspberry Pi processor prior to it being mounted. The instrument itself was mounted on a rotating turntable in order to facilitate scanning. Figure 2c shows the raw output from imaging the CFL for calibration purposes. The image exhibited the smile spectral artefact. The result of post-processing for smile removal is shown in Figure 2d. The discrete spectral peaks in the blue, green and red portions from the CFL are visible. In the case of the CFL, processed images were used for spectral calibration and other processed images were used for spectral analysis and mineral identification.

### 2.6. Sample Processing

**Figure 3:**
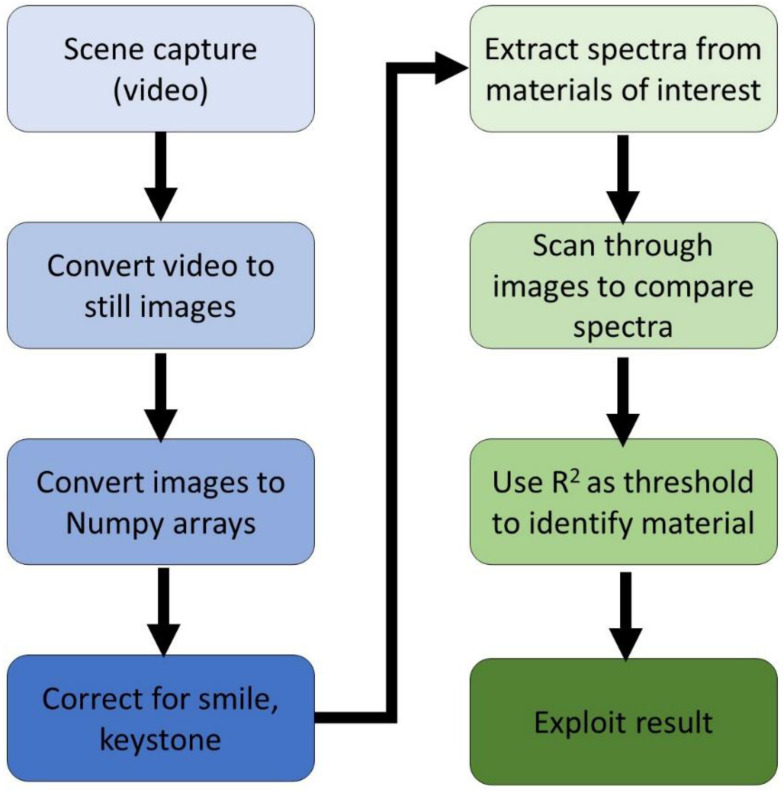
High level overview of the hyperspectral sensor image processing workflow from capturing the video to exploiting the processed result.

Figure 3 provides a high-level overview of the pushbroom sensor operation and data processing.

The output from the pushbroom sensor was an 8-bit RGB video file (MP4 format for the RPi cameras, AVI for the ZWO camera) which was processed to a series of still images of uncompressed JPEG format using open source-image processing software based on Pillow (PIL) and Numpy Python libraries. ImageJ was also used to assist with the analysis of imagery [52]. While a linearized raw pixel format would be desired, the use-case of such sensors obviates minimizing storage, and the sensor image processing pipeline provides these compressed formats by default and we show that this compression does not impact spectral identification. Following the capture of the scene and still image processing by the pushbroom sensor, additional processing was required to assist with calibration and the treatment of additional sensor-related effects. Hyperspectral systems are subject to spatial and spectral aberrations that degrade the critical image registration needed for spectral analysis, notably smile and keystone. Smile occurs due to a change in the central wavelength with slit height. This appears as bent spectral lines where the position of the spectral peak will change with increasing distance above or below the center position of the slit (example shown in Figure 2c) [53]. Keystone causes a positional change of pixel with wavelength, resulting in misregistration of 2D images of differing wavelengths during attempts to create a hyperspectral image cube [54].

Smile and keystone were removed through the additional use of Numpy and PIL Python image processing libraries. Image skew was used to reverse the smile distortion, using the discrete spectral peaks of the CFL as a ready reference for smile removal. The image skewing functionality was able was able to preserve pixel brightness levels over the image that would otherwise have occurred through spreading pixels in the correction. Keystone was removed through cropping sequential images in the hyperspectral cube to re-register the stack. Increasing the cropping amount as a function of wavelength up to a total of 15 pixels for the 600–800 nm range was sufficient to remove the mis-registration caused by keystone.

Raw imagery and video were considered as formats for the sensor output processing in order to maximize image quality [32]. While the sensors were capable of outputting raw outputs, consideration was given to onboard storage and processing constraints, particularly for space-based applications. Radiation damage and degradation to electronics and storage devices, particularly open-source Raspberry Pi hardware, can occur through continued exposure or space weather events^58^. These factors place constraints on component lifetimes and memory availability for on-board processing. In addition, cubesat-sized space missions possess significant bandwidth constraints, limiting the amount of data that can be downlinked during each satellite pass [55]. In order to address these limitations, while still allowing the use of low-cost electronics in the space environment, this research focused on sensor operations that would minimize processor and memory usage, as well as reducing as much as possible the data required to be downloaded. It was also noted that the RPi Mk 1 and Mk 2 cameras, while able to capture still images in raw format, raw video capture would greatly reduce the framerate and would be unsuitable for a pushbroom sensor. This motion was required in order to pan the sensor across the scene. In this way, a hyperspectral cube was able to be generated. Initial field trials using raw images made creation of a hyperspectral cube impractical. This forced the use MP4 video and uncompressed JPEG in lieu of raw data formats. It also reduced the probability of image processing failure due to radiation-induced upsets, while at the same time identifying whether MP4 and JPEG-converted data would still return scientifically useful results.

The amount of light available to the sensor and the ability to distinguish spectral phenomenology above sensor noise though the dispersive optics was also a consideration for designs in this work. The amount of energy available to each pixel is a function of variables provided by:

E_s_ = S_f_ M_r_ L_s_ Q_e_ G_e_ (2)

where E_s_ is the flux available at the sensor calculated from Formula (2), S_f_ is the total solar flux available in the sensor’s environment, M_r_ is the portion of energy reflected by the mineral of interest (provided as the worst case of 600, 700 and 800 nm wavelengths), L_s_ is the portion of energy passed by the sensor optics, Q_e_ is the quantum efficiency of the sensor and G_e_ is the portion of energy passed by the slit, representative of the worst case for dispersive optical designs.

A review of published spectral curves of the materials of interest investigated in this work [56,57] revealed that hematite at 600 and 700 nm possessed the lowest percentage of reflected energy (as little as 5% at 600 nm). This value was chosen to present a worst-case reflection case and used as a minimal reflectance value to assess sensors responses to low light conditions.

Other sensor properties affecting availability of photon energy are shown in Table 2. The absorption aspects of the Bayer filter where present are shown in a separate column.

The objective lens diameter of 30 mm and focal length of 80 mm was used in this analysis. The presence of a Bayer filter on the sensor was also identified as a factor affecting sensor energy transmission through the instrument. Both RPi cameras possess a Bayer filter as default options and the effect of the filter on incident energy needed to be considered [23,32]. Quantum efficiency Q_e_ per wavelength for the Bayer filtered sensors was 5–15% lower than for the non-Bayer sensor type (Table 2) and possessed higher absorption areas at the intersection of each band (blue/green, green/red).

The effects of a potential diffraction grating were considered on the wavelengths of interest. An Edmund Optics 600 lines/mm transmission grating with a 28.7° blaze angle was used for this analysis (Edmundoptics.com, accessed on 26 June 2021). The grating diffraction efficiencies for each wavelength are shown in Table 2. The width of the entrance slit was used to represent a worst-case transmission energy trade. Slits have typically been used with dispersive spectral sensors; the objective optics must be designed to match their width on the order of micrometers.

The expected transmission percentages referenced to a white Lambertian target were compared with actual results obtained from the hematite sample (experiment setup shown in Figure 1). These were then compared with the sampled pedestal dark frame current obtained when capturing the spectra for each sensor as described in Equation (1). The dark frame current was obtained through sampling the portions of the image frame where the spectra were absent at 20 °C. This equated to the upper and lower 20% of the image frames.

The default settings and automatic adjustment of the black level in each sensor rejected the majority evidence of pedestal dark current and noise. The user must adjust these settings to avoid losing signal at the balance of including noise. Dark frames were examined to confirm similar noise histograms to those published research [32] at different black level settings, by exploiting the raw Bayer capture software and sensor control bus. Part of this research was to determine whether the truncation of noise values would preclude the sensors’ ability to return usable signal, particularly for the hematite sample (Table 2, Figure 1).

### 2.7. Comparison and Discrimination Methods

How do the spectral outputs of hyperspectral sensors developed from these low-cost cameras compare against laboratory-grade instruments? To investigate this criterion and following post-processing to remove smile and keystone, open-source software was used to extract the spectra from the samples over the 600–800 nm ferric response wavelengths (high-level overview in Figure 3). To enhance robustness and provide consistency, 50 spectral measurements per sample were extracted and averaged. These spectra were then compared with curves obtained from the samples using a laboratory grade Spectral Evolution SR-3500 spectroradiometer in sunlit conditions similar to those in which the pushbroom sensor operated. The SR-3500 spectral curves were considered in this work to be the benchmark against which the custom pushbroom sensors were compared. Spectra sampled by the SR-3500 were exploited by using proprietary software (Spectral Evolutions package DARWin SP v.1.2.5060).

In this process, consideration was given to the dynamic range limitations of the 8-bit sensors of the three tested cameras. This was particularly prominent when exposing for the highly reflective specular white card and lower reflective materials under direct sunlight (Figure 1). This meant that spectral brightness was not able to be compared due to the requirement to carefully control sensor exposures to between 80 and 90% of the sensor’s saturation values. Initial testing of the Mk 1 and Mk 2 Raspberry Pi cameras (Appendix A) revealed that automatic exposure times were able to derive suitable results, while initial trials of the ZWO Astrocam [31] required manual adjustment to the exposure. To facilitate comparison, the pushbroom sensor and SR-3500 spectral curves were normalized by dividing the result by the maximum reading. The pushbroom/SR-3500 spectral curve comparison was conducted by undertaking linear regressions comparing 41 values at 5 nm intervals between 600 and 800 nm. R^2^ values resulting from the linear regression tests between the custom spectrometers and SR-3500 were used to infer accuracy of measurements.

Are the low-cost sensors able to distinguish between different materials and identify materials of interest? Additional software processing was used to generate a spectral library of the samples that would be used to assess this criterion. The first phase of this work was to determine whether each pushbroom sensor was able to associate and group spectral readings from different parts of the same material or similar materials. Two rust samples, hematite and mudstone, were grouped into an iron oxide family; three olivine samples grouped to an olivine family and the two vegetation samples into a vegetation family. In order to achieve this, 10 spectral curves were obtained from different regions of each of the 15 studied materials. Multiple regression was performed to identify the variance between each of the spectra of the investigated sample. If the spectra were similar above a predetermined confidence interval represented by a set R^2^ value, the spectra of interest were determined by the algorithm to belong to a mineral family and not of different composition (for example vegetation versus olivine). For the purposes of qualitative comparison, the spectra returned from the SR-3500 and custom sensors were normalized. This was achieved by subtracting the lowest value to place the base of the curve at zero, and dividing this curve by the maximum value to provide an intensity range of 0–1.

To appraise and evaluate whether low-cost instruments could discriminate between individual minerals, we conducted an experiment to determine if the software were able to sift through the hyperspectral imagery on a row-by-row basis, comparing detected spectra with those contained in the custom spectral library. The comparison was performed by linear regression, with R^2^ used as a suitability coefficient. If a sampled spectrum passed a pre-determined R^2^ threshold, a bright pixel would be generated at that location. This sequential process then built up an image mask where the material of interest was represented by bright pixels, and undesired areas were represented by pixels of value zero. False detections (i.e., pixels that fell outside the mineral mask) were also collected and counted, and the ratio between the two used as an indication of success.

In order to gather statistically significant data, the similar material and discrimination trial processes were repeated with all sensors, but with the difference of including the addition of the blue-green components of the collected spectra to the spectral libraries of each instrument. This meant that, instead of restricting the tests to 600–800 nm, a larger spectral curve (435–800 nm) was available for the similar material and discrimination trials. The inclusion of this additional activity enabled analysis of the sensor’s performance with the increased spectral range and identified how the full spectra affected the instrument’s ability to identify material and material families as compared to their performance in the VIS/NIR component of the spectrum.

## 3. Results

### 3.1. Dark Frame Output and Spectral Comparisons

Table 3 shows minimum, average, maximum and standard deviation of DC pedestal dark frame sensor output as measured by DN for each of the three cameras as derived from Equation (1). It also shows returned DN from analysis of the Hematite sample, inferred to be the lowest reflective sampled material (Section 2.5, Figure 1). The ZWO Mini possessed the highest average DN of the three sensors, with the RPi Mk 2 camera returning the lowest. Average DN measurements at 50 nm intervals identified the ZWO camera to be most sensitive to the spectral phenomenology of the Hematite sample, while the values of both RPi cameras were comparable.

**Figure 4:**
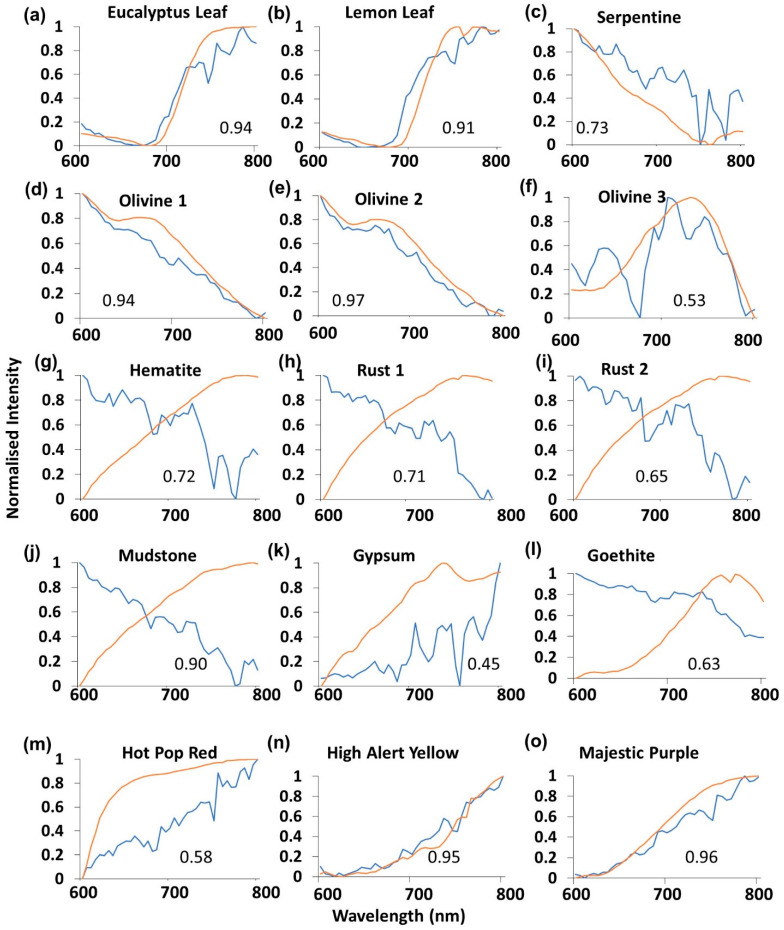
(**a**–**o**) RPi Mk 1 camera sensor (blue) normalized intensity curve graphical comparisons with the SR-3500 spectroradiometer (orange) for each of the sampled materials. R^2^ values for the fitted relationships are also shown.

Normalized spectral plot comparisons (Figure 4a–o) between the RPi v1Mk 1 camera sensor (blue) and SR-3500 (orange) showed small-scale variations, and R^2^ values ranged between 0.45 (Figure 4i) and 0.97 (Figure 4e). The shapes of the Mk 1 curves varied between samples, though qualitatively appeared similar between mineral class (vegetation, Figure 4a,b; olivine, Figure 4d–f; iron oxide, Figure 4g–i). The Mk 1 camera spectral curves fitted most closely with two vegetation samples (Figure 4a,b), two of the three olivine samples (Figure 3e,f) and two of the three color swatches (Figure 4n,o). The Mk 1 camera sensor spectral returns differed most greatly from the SR-3500 in the Olivine 1 (Figure 4d), Gypsum (Figure 4k), Goethite (Figure 4l) and Hot Pop Red (Figure 4m) samples. All of the iron oxide-related mineral samples (Figure 4g–j) exhibited decreasing NIR responses in the iron oxide samples when compared to the SR-3500. High R^2^ values are possible here due to the apparent reversed slopes, caused by the drop in signal when Bayer filters are present.

**Figure 5:**
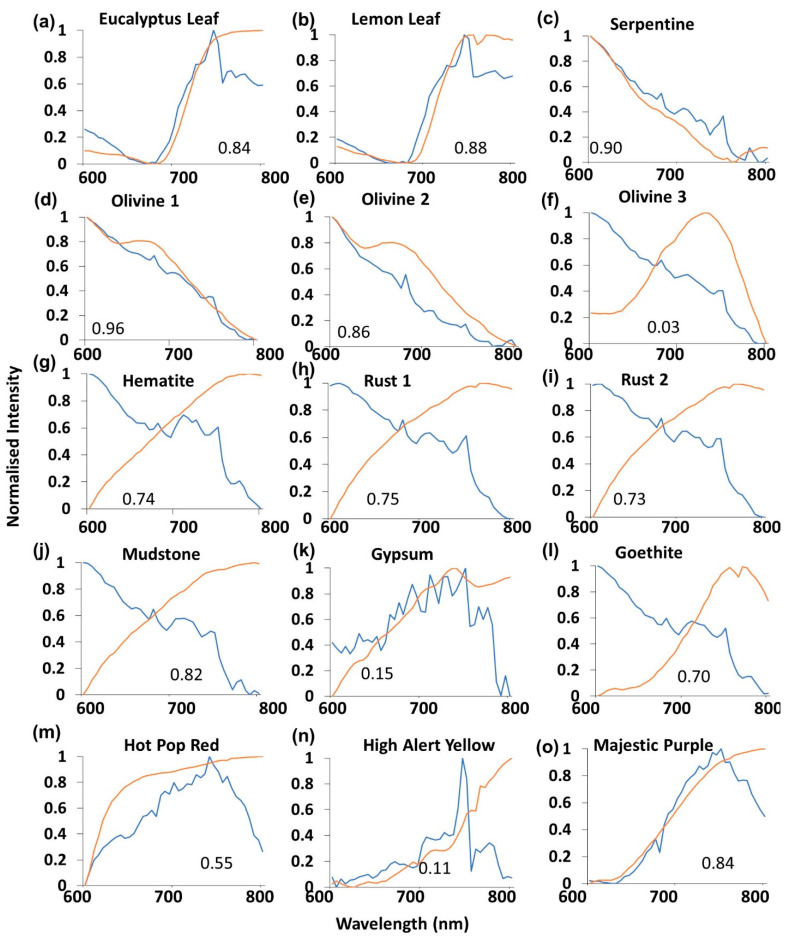
(**a**–**o**) RPi Mk 2 camera sensor (blue) normalized intensity curve graphical comparisons with the SR-3500 spectroradiometer (orange) for each of the sampled materials. R^2^ values for the fitted relationships are also shown.

Spectral results comparisons (Figure 5a–o) between the Mk 2 camera sensor (blue) and SR-3500 instrument (orange) showed small-scale variations, and R^2^ values ranged between 0.03 (Figure 5f) and 0.96 (Figure 5d). Spectral results were similar to the Mk 1 (Figure 4) with similar reductions in the NIR portion of the spectrum for the materials containing iron oxide (Figure 5g–i). Substantial differences in spectral return between the Mk 1 and Mk 2 instruments were present in the High Alert Yellow sample, where NIR spiked and decreased sharply (Figure 5n). This did not occur in the equivalent Mk 1 sample (Figure 5n). Gypsum and Goethite samples from the Mk 2 sensor more closely fitted with the SR3500 readings (Figure 5k,l) when compared with the equivalent Mk 1 samples (Figure 4k,l). The Mk 1 outperformed the Mk 2 camera sensor on the Eucalyptus Leaf, Hot Pop Red, High Alert Yellow and Majestic Purple materials. In the Majestic Purple material analysis, the Mk 1 camera substantially outperformed the Mk 2 with the correct shape (R^2^ 0.96, Figure 4o cf. 0.84, Figure 5o).

**Figure 6:**
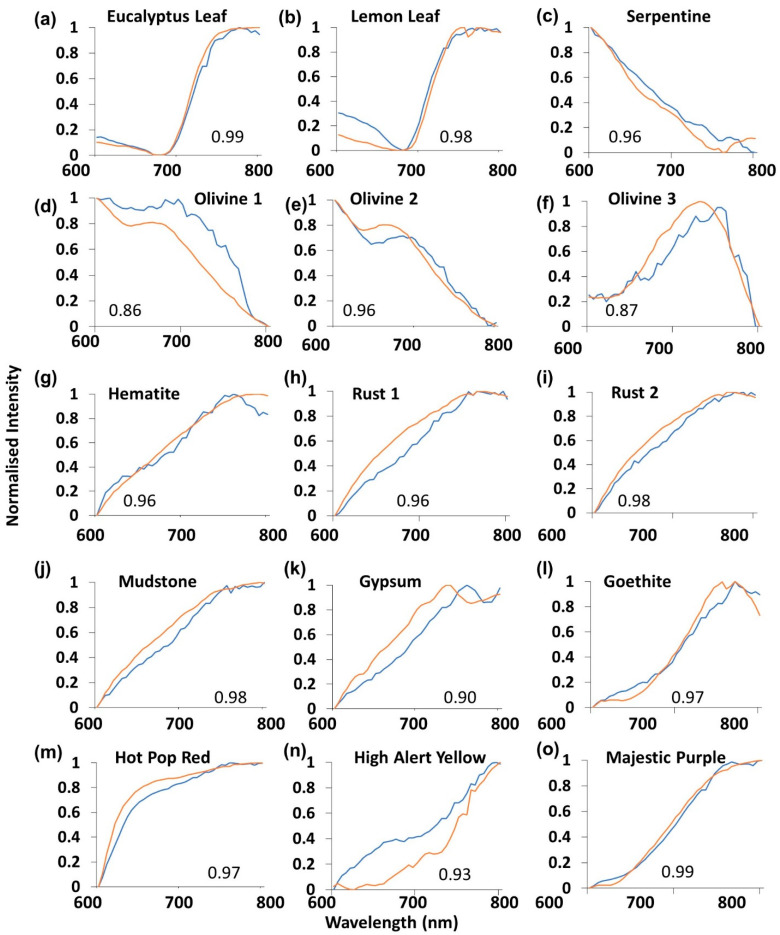
(**a**–**o**) ZWO camera sensor (blue) normalized intensity curve graphical comparisons with the SR-3500 spectroradiometer (orange) for each of the sampled materials. R^2^ values for the fitted relationships are also shown.

The spectral curves of the ZWO sensor (blue, Figure 6a–o) more closely agreed with those of the SR-3500 (orange) when compared with the Mk 1 and Mk 2 sensors (Figure 4 and Figure 5). R^2^ values ranged between 0.86 (Figure 6d) and 0.99 (Figure 6a,o). With the exception of the Olivine 3 sample (Figure 6f), the spectral curves for the ZWO instrument showed fewer small-scale variations when compared with the Mk 1 and Mk 2 (Figure 4 and Figure 5). The monochrome sensor outperformed the other two sensors in all materials with the exception of High Alert Yellow, Olivine 1, and Olivine 3 (Figure 4, Figure 5 and Figure 6d,f,n).

### 3.2. Material Detection Analysis

Table 4 shows the results from performing regression analysis on similar materials. R^2^ values for the Mk 1, Mk 2 and ZWO sensors ranged between 0.98 and 0.99, 0.96 and 1.0 and 0.98 and 1.0, respectively. The ZWO camera sensor possessed higher R^2^ values across all sampled materials on average (0.993, ZWO cf. 0.989, Mk 1 and 0.994, Mk 2). Variations between values were higher with the Mk 2 sensor (standard deviation of 0.0084) than for the Mk 1 (standard deviation of 0.0059) and ZWO sensor (standard deviation of 0.0059).

**Figure 7:**
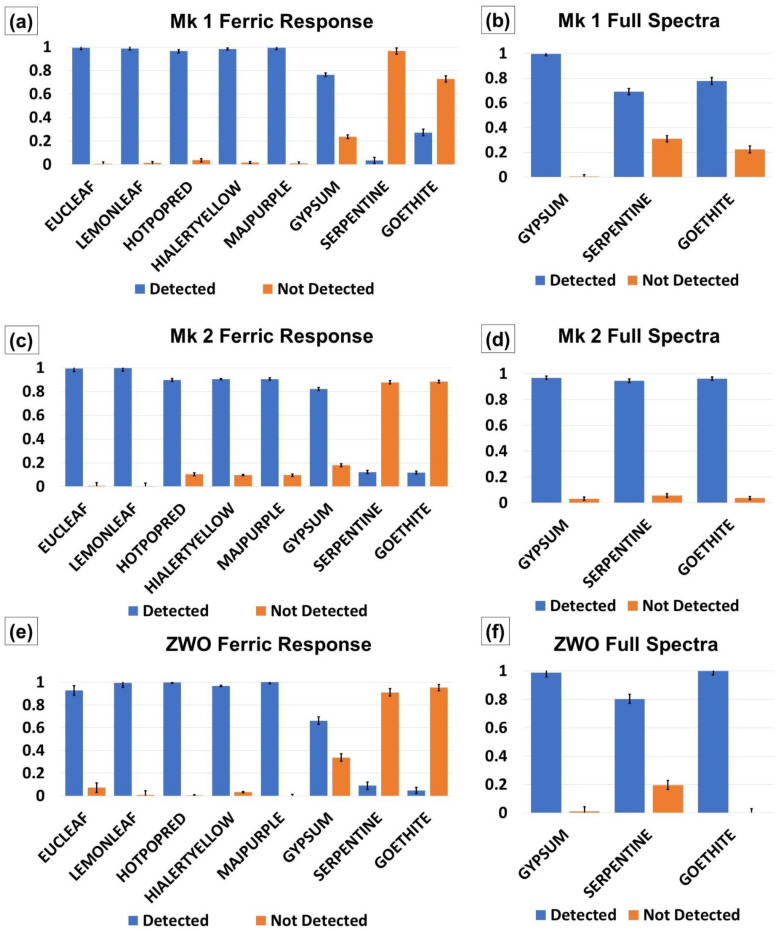
(**a**). RPi Mk 1 ferric response spectra discrimination analysis of non-family materials. Actual detections (blue) and false detections (orange) are shown. (**b**) RPi Mk 1 full spectrum discrimination analysis Gypsum, Serpentine and Goethite materials. (**c**) RPi Mk 2 ferric response spectra discrimination analysis of non-family materials. Actual detections (blue) and false detections (orange) are shown. (**d**) RPi Mk 2 full spectrum discrimination analysis Gypsum, Serpentine and Goethite materials. (**e**) ZWO sensor ferric response spectra discrimination analysis of non-family materials. Actual detections (blue) and false detections (orange) are shown. (**f**) ZWO full spectrum discrimination analysis for Gypsum, Serpentine and Goethite materials with actual (blue) and false (orange where present) shown.

Figure 7a–f shows the results of the individual material discrimination analysis from the RPi Mk 1, Mk 2 and ZWO camera pushbroom sensors. Error bars display the average standard deviation between the 50 spectral measurements obtained for each sample as described in Section 2.7. The sensor performance when identifying the tested color swatches and two types of leaf samples (Figure 7a,c,e) was successful for all of the tested sensors. The Eucalyptus Leaf was able to be identified, and discriminated from the Lemon Leaf sample for the RPi Mk 1 and Mk 2 sensors (Figure 7a,c). The ZWO sensor exhibited higher false detections in the Eucalyptus Leaf sample where portions of the Lemon Leaf were identified as Eucalyptus Leaf samples.

Mk 2 discrimination analysis of the Gypsum sample (Figure 7c) was subject to more false detections when compared to the Mk 1 results (Figure 7a). The ZWO sensor also exhibited higher false detections than the Mk 1 camera for the Gypsum sample (Figure 7e). Detection of the Goethite and Serpentine samples was less successful across the three sensors (Figure 7a,c,e). False detections occurred in other materials including the Grey Card, Mudstone, Hematite and Olivine samples. The Mk 2 camera provided the best true-to-false detection ratio for Serpentine of all sensors (Figure 7C), however, the level of false detections (orange, Figure 7c) exceeded those of true detections (blue, Figure 7c).

Full-spectrum detections of the three non-family minerals (Gypsum, Serpentine, Goethite) revealed significant improvement in the ratio of true to false detections of all three trialed sensors (Figure 7b,d,f). The average of true detections for the Mk 1 and Mk 2 sensors was 0.82 and 0.96, respectively (false detections being 0.18 and 0.04, respectively), and the ZWO the average true detection was 0.93 (Figure 7f). While the Goethite sample for this sensor exhibited no false detections, and the Gypsum sample outperformed the other sensors (0.99 ZWO, Figure 7f compared with 0.98 Mk 1 and Mk 2, Figure 7b,d), the Serpentine sample exhibited reduced spectral detection performance when compared with the RPi Mk 2 sensor (0.80 ZWO compared with 0.94, Mk 2, Figure 7b,d,f).

**Figure 8:**
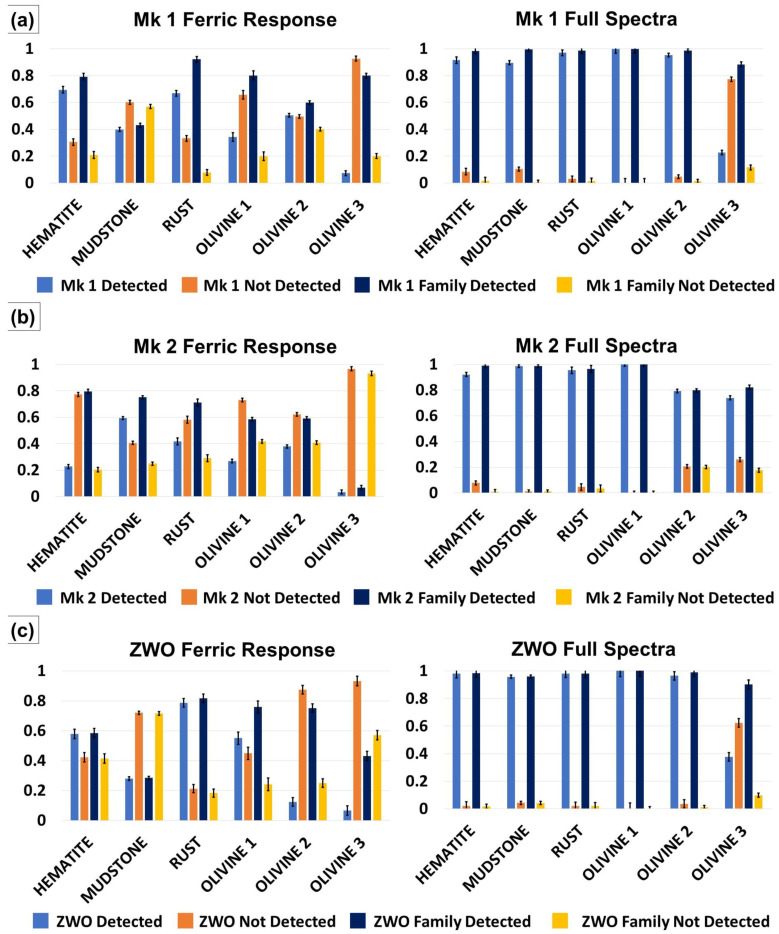
(**a**). RPi Mk 1 ferric response spectra (left) and full-spectra (right) discrimination analysis of the iron oxide and olivine mineral families including single mineral detection (Mk1 Detected), single-mineral false detection (Mk 1 Not Detected), mineral family detection (Mk 1 Family Detected) and mineral family false detection (Mk 1 Family Not Detected). (**b**) RPi Mk 2 ferric response spectra (left) and full-spectra (right) discrimination analysis of the iron oxide and olivine mineral families including single mineral detection (Mk 2 Detected), single-mineral false detection (Mk 2 Not Detected), mineral family detection (Mk 2 Family Detected) and mineral family false detection (Mk 2 Family Not Detected). (**c**) ZWO ferric response spectra (left) and full-spectra (right) discrimination analysis of the iron oxide and olivine mineral families including single mineral detection (ZWO Detected), single-mineral false detection (ZWO Not Detected), mineral family detection (ZWO Family Detected) and mineral family false detection (ZWO Family Not Detected).

Figure 8a–c shows the mineral family ferric response (left) and full-spectra (right) detection and false detection analysis for the iron oxide (Hematite, Mudstone, Rust) and olivine (Olivine 1, Olivine 2 and Olivine 3) families. Error bars display the average standard deviation between the 50 spectral measurements obtained for each sample as described in Section 2.7. For all 600–800 nm and full-spectrum detections, the family-based detection ratios were consistently equal to or exceeded the detection/false detection ratios of the individual mineral analysis (Figure 8a–c). The Mk 1 sensor 600–800 nm results indicated that this instrument was best able to detect the Hematite and minerals (Figure 8a, left). The Mk 1 sensor was better able to detect minerals belonging in families, with a slightly better result for the Olivine family (average 0.73) than the iron oxide family (average 0.71). The full-spectra analysis of the family materials revealed a significant increase in true (average Mk 1 Detected) to false (Mk 1 Not Detected) detections (Figure 8a) for most minerals. The exception was the Olivine 3 sample, which exhibited lower true detections than the other olivine samples (0.22, Olivine 3 compared with 1.0 Olivine 1 and 0.95, Olivine 2, Figure 8a). The Olivine 3 true detections were significantly higher in the family detections, indicating the RPi Mk 1 sensor successfully identified the mineral as belonging to the Olivine family. Average true detections for the olivine and iron oxide families were 0.96 and 0.99, respectively.

Family detections for the Mk 2 sensor are shown in Figure 8b (600–800 nm spectra sampling on the left, full spectra on the right). The Mk 2 sensor exhibited poorer 600–800 nm detections when compared with the Mk 1 sensor (Mk 2 average 0.32, Figure 8b, Mk 1 average 0.44, Figure 8a). The Mudstone sample returned the highest true detection result (0.59, Figure 8b) and the Olivine 3 sample the lowest (0.03, Figure 8b). Mineral family true detections were consistently higher than the individual detections for the Mk 2, but lower overall than for the Mk 1 sensor (Mk 2 average 0.59, Figure 8b; Mk 1 average 0.71, Figure 8a). The Hematite sample exhibited the highest true family detections (Mk 2 Family Detected, Figure 8b), and Olivine 3 the lowest (Mk 2 Family Detected, Figure 8b). Full-spectra analysis revealed a similar increase in true detections for the mineral families though were higher overall than those of the Mk 1 (Mk 2 average 0.90, Figure 8b compared with Mk 1 average 0.83, Figure 8a). The Olivine 1 detections were highest for the Mk 2 full spectra analysis, and the Olivine 3 detections the lowest (Figure 8b). Mineral family detections for the Mk 2 sensor (average 0.93, Figure 8b) were slightly lower than those for the Mk 1 (average 0.97, Figure 8a).

The overall ZWO sensor mineral family 600–800 nm true detection analysis was higher than the Mk 2 sensor but lower than the Mk 1 or the Mk 2 sensor (ZWO average 0.40, Figure 8c; Mk 2 average 0.32, Figure 8b; Mk 1 average 0.44, Figure 8a). The Rust, Olivine 1 and Olivine 2 exhibited the highest true detections, and Olivine 3 the lowest. The family-based detections for the ZWO sensor indicated a better performance overall than the Mk 2 sensor (average ZWO family detections of 0.60 compared with average Mk 2 family detections of 0.58; Figure 8b,c) but lower than that for the Mk 1 sensor (average ZWO false detections of 0.60 compared with average Mk 1 family detections of 0.72; Figure 8a,c). The ZWO family detection analysis performed slightly better than for the individual detections (average 0.56 family compared with 0.54 individual, Figure 8c). The Rust and Olivine 1 samples returned the highest family detections (ZWO family detections of 0.82 and 0.76, Figure 8c). The Olivine 3 sample returned the lowest true detections from the ZWO sensor. As with the other sensors, full-spectra detections for the ZWO showed a high increase in true detections compared to false detections for all individual-sampled minerals with the exception of Olivine 3 (Figure 8c). Excluding Olivine 3, ZWO detections ranged from 0.96–1.0. The Olivine 3 sample revealed a true detection ratio of 0.38, bringing the total average to 0.88, within the same range as the Mk 1 sensor average (0.83, Figure 8a) and Mk 2 (0.90, Figure 8b).

Mineral family-based detection analysis also showed an increase in true detection ratios for all mineral types (ZWOFD, Figure 8c). The average family detections for the ZWO sensor were higher than those of the Mk 2 and equivalent to the Mk 1 (ZWO family detections of average: 0.97, Figure 8c; Mk 2 Family Detected average: 0.93, Figure 8b; Mk 1 family detections of average: 0.97, Figure 8a).

## 4. Discussion

### 4.1. Overview and Major Findings

A pushbroom sensor built from three of the five consumer-grade imaging sensors identified from a tailored system engineering trade study were tested using materials exhibiting spectral phenomenology in the 600–800 nm region. While previous research has investigated pushbroom sensors [11,13,27], this work used comparative analysis with a laboratory-grade SR-3500 to quantify the performance of these sensors. These data were then used to determine the utility of each sensor for VIS/NIR spectral applications.

Low-Cost hardware and open-source Python software were deliberately chosen to maximize the stakeholder base for such instruments among low-budget users and citizen scientists. Additionally, the readily available software negates the requirement to navigate proprietary licensing and allows for installation on diverse platforms such as the Raspberry Pi and other single board computers.

The noise analysis shown in Table 4 indicates that all cameras were able to return a useful signal above the noise floor, including for Hematite which was the mineral exhibiting the lowest reflectance values of all materials under study (Table 3). While raw Bayer image capture from the RPi cameras has been used for dark frame assessments in previous research [32], the inability to produce video with a frame rate useful for pushbroom sensors meant that this approach was impractical. It was found that the NIR secondary transmissions in the Bayer filter of the Mk 1 and Mk 2 cameras, particularly in the blue and to a lesser extent red channel, provided sensitivity beyond 700 nm for both these sensors.

Spectral analysis across the 15 sampled materials revealed a diversity in fitted relationships with samples when compared to the SR-3500 spectroradiometer. The two vegetation samples (Lemon Leaf and Eucalyptus Leaf, Figure 1) exhibited high R^2^ values across all three sensors (Figure 4, Figure 5 and Figure 6a,b). Vegetation exhibits a distinctive spectral curve with significant differences between the red and NIR portions of the spectrum [58]. This characteristic difference in the 600–800 nm portion of the spectrum enabled vegetation curves to be readily fitted with high R^2^ values with the SR-3500. These sensors may find utility in vegetation analysis. The Majestic Purple and Olivine 2 samples (Figure 1) also exhibited high R^2^ values (Figure 7a) across all three sensors and possessed high contrast in values between the red and NIR spectral values (Figure 4, Figure 5 and Figure 6e,o). This trend did not occur in other samples exhibiting similar contrasts in spectral reflectance, such as the other olivine or iron oxide-related samples. There were differences between R^2^ values between the sensors for these samples (Figure 4 and Figure 5). Assessment of spectral performance revealed the ZWO sensor to be the best performing sensors over the RPi cameras. The ZWO camera was advantageous in that it did not possess a Bayer filter, which reduced the available light for the sensor. In addition, the need to use MP4 video for the Mk 1 and Mk 2 sensors meant the frames were pre-processed with on-board software, affecting the white balance and gain of the output. This pre-processing has favored the green channel of the camera, de-emphasizing the other bands where red and NIR information are captured [26,41]. Furthermore, it was found that absorption troughs were present in the NIR (700–800 nm) portion of the returned white specular reflection spectrum for the Mk 1 and Mk 2 cameras. These troughs affected spectral returns and the suitability of the fitted relationship varied in accordance with where the spectral peak of the sampled mineral occurred in relation to the position of the troughs. In contrast the ZWO sensor returned a smoother, more consistent result over the 600–800 nm spectral range. The blue channels of the Mk 1 and Mk 2 were found to contribute primarily to the NIR signal beyond 700 nm for the Mk 1 and Mk 2 cameras. Signal output from the red channel decreased beyond 700 nm and the interactions between these Bayer filter channels have affected the spectral returns from these cameras. Thus, while the production of color imagery made the data more human readable and intuitively easier to work with [11,26], the absorption limitation of the Bayer filters and inability to capture raw video was found overall to affect the spectral performance of the Raspberry Pi cameras.

The complementary nature of the RPi camera curves versus the SR-3500 spectroradiometer for the iron oxide samples (Figure 4 and Figure 5g–k) was caused by the low signal to noise ratios and pre-processing of these cameras in the NIR band. All of these materials were poorer reflectors (Table 3), decreasing the amount of information available for the sensors. Additionally, the size limitation of the on-board lens for the Raspberry Pi v2, as compared to a dedicated s-mount lens available for the Raspberry Pi v1 camera, likely contributed to its poorer spectral performance when compared to the other two sensors. Thus, sensor optic considerations were identified as a performance dependency, along with resolution of the sensor itself and automatic pre-processing.

These findings were verified through the capture of raw, unprocessed still images for the Mk 1 and Mk 2 cameras. While not possible to generate spectral image cubes, the raw imagery bypassed the pre-processing of the MP4 video, allowing a better representation of the sample spectra. This method enabled results from the RPi cameras to be more consistent when compared to the ZWO 3500. In light of these findings, RPi cameras are better suited to vegetation analysis or transmission spectral or multispectral applications [25,43], with the ZWO sensor much better suited for reflective spectral analysis.

Results from the similar materials test revealed that the Raspberry Pi Mk 2 camera possessed the highest R^2^ values on average, with the Mk 1 the lowest (Table 4). The overall high R^2^ values (>0.96, Table 4) from all of the sensors indicated all of these cameras would be able to identify families of materials, such as iron oxide, olivine, or vegetation. The presence or absence of a Bayer filter, or difference in resolution appeared not to have an appreciable difference in the similar materials test. Instead, this test showed the consistency of the spectral curve was more important than its spectral accuracy as revealed by comparison with the SR-3500. This was found useful in the material discrimination test, where regression was used to compare materials with a custom spectral library in per-pixel analysis, and potential detection of material families was tested. All sensors were readily able to detect the color swatches and vegetation samples with few false positives (Raspberry Pi Mk 1, Figure 7a, Raspberry Pi Mk 2, Figure 7b, ZWO camera, Figure 7c).

The Mk 1 sensor was able to identify the Gypsum mineral sample, Rust and Hematite minerals and iron oxide families, and Olivine 1 and Olivine 3 as belonging to the Olivine mineral family. Other minerals exhibited higher false detections (Figure 7a and Figure 8a). The Mk 2 instrument was able to detect the Gypsum material and associate the Hematite and Mudstone materials with the iron oxide mineral family, but also suffered from higher false positives with the other mineral tests (Figure 7b and Figure 8b). The 600–800 nm performance of the ZWO instrument fell between that of the Mk 1 and Mk 2 sensors for individual and mineral-family detections (Figure 7c and Figure 8c). This sensor best performed in the identification of Rust and Olivine 1 materials belonging to the iron oxide and olivine mineral families, respectively.

While the panchromatic camera spectral output was more similar to a laboratory-grade instrument (Figure 6a–o), the results from the material discrimination analysis indicated that the use of a monochrome sensor was not a significant driver for distinguishing between material spectra (Figure 7 and Figure 8). Successful detection results depended more on the pre-acquisition of spectra in a spectral library and the properties of the material under investigation. For the Mk 1 and Mk 2 sensors, this library would account for the results returned from these cameras, while the ZWO spectra were more similar to those produced by the SR-3500 spectroradiometer (Figure 6). The 600–800 nm detection results indicated that these instruments were more suited to vegetation and artificial material discrimination, as suggested by the color swatch, Lemon Leaf and Eucalyptus Leaf results (Figure 7a,c,e).

The removal of the 600–800 nm constraint to allow the pushbroom sensors to use the full spectra in the detection analysis vastly improved the performance of all trialed instruments (Figure 7b,d,f; Right hand graphs in Figure 8a–c). This indicates that for discrimination of minerals exhibiting more subtle phenomenology in the 600–800 nm portion of the spectrum, the use of the full spectrum gathering capabilities (435–800 nm) of these instruments would be preferred. The tradeoff for this advantage was a doubling of spectral sampling required (435–800 nm compared with 600–800 nm) and subsequent increase in processing time that a deployed configuration would have to accommodate.

### 4.2. Other Sensor Considerations

Apart from spectral and material discrimination considerations, the three sensors differed in their physical attributes. The Raspberry Pi Mk 1 and Mk 2 cameras were able to be housed in a 30 mm long attachment to the pushbroom sensor, whereas the ZWO extended the size of the pushbroom sensor by 100 mm. Additionally the Mk 1 and Mk 2 cameras when configured for the pushbroom sensor weighed 7 g and 4 g, respectively, while the ZWO sensor weighed 100 g. These physical differences raised implications for size and weight restrictions in constrained environments, such as a high-altitude balloon or cubesat payloads, where size and weight are critical considerations. The ZWO camera would exceed a 2 U cubesat space (10 × 10 × 20 cm) in order to fit a pushbroom sensor made from it, whereas a pushbroom sensor made from the Raspberry Pi sensors could be constrained within a 2 U (10 × 10 × 20 cm) cubesat volume [29]. The choice of sensor for the pushbroom also forced consideration for the type of microprocessor board required to operate the sensor. While not subject to quantitative analysis in this work, it was found that required processor units varied substantially in size and capabilities. The volume of a Raspberry Pi Zero is substantially less than that required for the LattePanda board used to operate the ZWO camera. Future work will be conducted to determine whether smaller processor boards are able to successfully control the ZWO camera, thus reducing required size constraints.

### 4.3. General Considerations and Future Work

The trialed pushbroom sensors were able to distinguish between families of materials, though the ZWO sensor substantially outperformed the two Raspberry Pi cameras for spectral accuracy when compared to the SR-3500. While a custom spectral transformation library would probably be required to correlate Raspberry Pi spectra with material types, all sensors could be used to assist with VIS/NIR mineral identification and classification. The spectral responses for vegetation were particularly successful in the spectral and discrimination trials for all tested sensors, indicating that this pushbroom sensor would be ideal for field vegetation type and health classification [59].

Consideration was given to the repeatability of these measurements with the trialed camera systems. The ability to repeat these results will be dependent on ensuring identical sensor design, using similar environmental conditions and sensor type. Each of the cameras used has demonstrated utility in amateur astronomy or remote sensing applications [14,24,26,31,32]. The RPi Mk1 camera has previously produced repeatable results in multispectral and hyperspectral applications [14,24,26] while extensive laboratory experimentation with the Mk2 camera demonstrated its consistency over multiple units [32]. Can the results be repeatable in the field? As described in Section 2.5, these experiments were conducted under direct sunlight to ensure consistency of light source. Considerations for field trials with these sensors will include controlling, or at least accounting for, the effects of external factors. The trials in this work were conducted under controlled lighting and positioning conditions to ensure consistent illumination of the samples and minimize atmospheric effects. This ensured repeatability of results. Field work will be subjected to more variation. Atmospheric effects, such as scattering and dust [60] will impact spectral accuracy. Both Earth and Mars atmospheres exhibit these properties [61]. Minerals may also be obscured in natural environments, or mixed with other mineral types. An example is the iron oxide dust prevalent over much of Mars, covering many underlying materials in an obscuring blanket [61]. Future trials will be conducted using drones and stratospheric balloon flights in order to further characterize these sensors for deep space remote sensing [62]. The results obtained in this work will be used as a best-case scenario from which to compare future field results. Future work will also examine an absolute optical link budget study for the next iteration of instruments.

The MastcamZ instrument on the Mars 2020 rover is planned to provide unprecedented multispectral imagery of the Red Planet’s surface [63,64]. The MastcamZ instrument possesses similar sensitivity to the VIS/NIR wavelengths studied in this work, though these wavelengths have been traditionally favored for vegetation analysis [2,4,24]. The MastcamZ, as with many cameras flown to Mars, was calibrated using materials of a known radiance comprising a target that is being carried on board Mars 2020 [64]. Results returned from the sensors tested here will be compared with those returned from comparable minerals identified by MastcamZ at Jezero Crater. This will entail field trips to identified Mars analogue sites in central Australia. Future analysis will also include customization of the spectral library pipeline to identify minerals of interest by developing a dedicated spectral library from the returned MarstcamZ imagery.

## 5. Conclusions

Three commercial-grade sensors were used to trial a diffractive pushbroom sensor design against materials exhibiting spectral properties in the 600–800 nm range. This was conducted in order to identify the utility of popular Raspberry Pi cameras and a low-cost astrophotography camera for investigating materials that exhibit VIS/NIR reflectance properties, and can be used in space analogue environments. While Raspberry Pi-based spectrometers were previously used for spectral applications, this work revealed that their utility for reflectance-based pushbroom imaging was limited. Conversely, the panchromatic astronomy camera-based sensor was much better suited for this application. All sensors were able to distinguish between different material, provided that a custom spectral library is generated for the Raspberry Pi cameras. These findings refine the utility and citizen science applications for these sensors, enabling intelligent choices for hyperspectral design. Future work will conduct field trials with these sensors in order to further characterize sensor utility.

## Figures and Tables

**Table 1:** Identified requirements for hyperspectral sensor.

Index	Requirement
1	The budget for the sensor shall not exceed AUD $1000.
2	The sensor shall be sensitive to at least the 600–800 nm VIS/NIR spectral range.
3	One sensor design shall include a Bayer filter.
4	One sensor design shall not include a Bayer filter.
5	The sensor and supporting electronics shall consume at most 15 W.
6	The sensor shall occupy a volume not exceeding 2 U (100 × 100 × 200 mm).
7	The sensor and supporting electronics shall weigh less than 1 kg.
8	The sensor design shall not require specialist assembly.
9	The sensor shall operate on an open-source, single-board computer.

**Table 2:** Environmental and sensor properties influencing available energy for the detector.

**Property**	**Value**		
**S_f_ at Earth Surface (600–800 nm)**	**295 Wm^−2^**		
**Mineral Reflectance**	**Hematite**		
Reflectance at 600 nm (%)	5		
Reflectance at 700 nm (%)	7		
Reflectance at 800 nm (%)	7		
**Sensor Q_e_**	**Monochrome**	**Rpi Mk1**	**Rpi Mk2**
Sensor Q_e_ at 600 nm (%)	78	50	78
Sensor Q_e_ at 700 nm (%)	65	50	50
Sensor Q_e_ at 800 nm (%)	40	40	45
Grating at 600 nm (%)	50		
Grating at 700 nm (%)	40		
Grating at 800 nm (%)	32		

**Table 3:** Minimum, average, maximum and standard deviation of dark frame sensor DN for each of the three cameras. Average DN of hematite spectral return between 600 and 650, 650 and 700, 700 and 750, and 750 and 800 nm is also shown.

Raspberry Pi Mk 1 Camera			
DN (Minimum)	DN (Average)	DN (Maximum)	DN (Std Deviation)
0	0.86	2.8	0.56
Hematite (DN, average 600–650 nm)	Hematite (DN, average 650–700 nm)	Hematite (DN, average 700–750 nm)	Hematite (DN, average 750–800 nm)
35	27	19	17
Raspberry Pi Mk 2 Camera			
DN (minimum)	DN (Average)	DN (Maximum)	DN (Std Deviation)
0	0.28	1.8	1.38
Hematite (DN, average 600–650 nm)	Hematite (DN, average 650–700 nm)	Hematite (DN, average 700–750 nm)	Hematite (DN, average 750–800 nm)
40	27	19	14
ZWO Mini Camera			
DN (minimum)	DN (Average)	DN (Maximum)	DN (Std Deviation)
0	0.73	1.8	0.24
Hematite (DN, average 600–650 nm)	Hematite (DN, average 650–700 nm)	Hematite (DN, average 700–750 nm)	Hematite (DN, average 750–800 nm)
46	41	34	26

**Table 4:** Mk 1, Mk 2 and ZWO sensor proximal mineral fitted relationships results.

Material	Mk 1 R^2^	Mk 2 R^2^	ZWO R^2^
Eucalyptus Leaf	>0.99	0.99	>0.99
Lemon Leaf	0.99	0.99	0.99
Olivine 1	0.99	>0.99	>0.99
Olivine 2	0.98	>0.99	>0.99
Olivine 3	0.98	>0.99	0.99
Rust 1	0.98	>0.99	0.98
Rust 2	>0.99	>0.99	0.98
Mudstone	0.99	>0.99	0.99
Hematite	0.99	>0.99	0.99
Gypsum	0.98	>0.99	>0.99
Goethite	0.99	1	>0.99
Serpentine	0.99	>0.99	>0.99
Hot Pop Red	>0.99	0.99	>0.99
High Alert Yellow	0.99	0.99	>0.99
Majestic Purple	0.98	0.96	0.98

**Figure A1:**
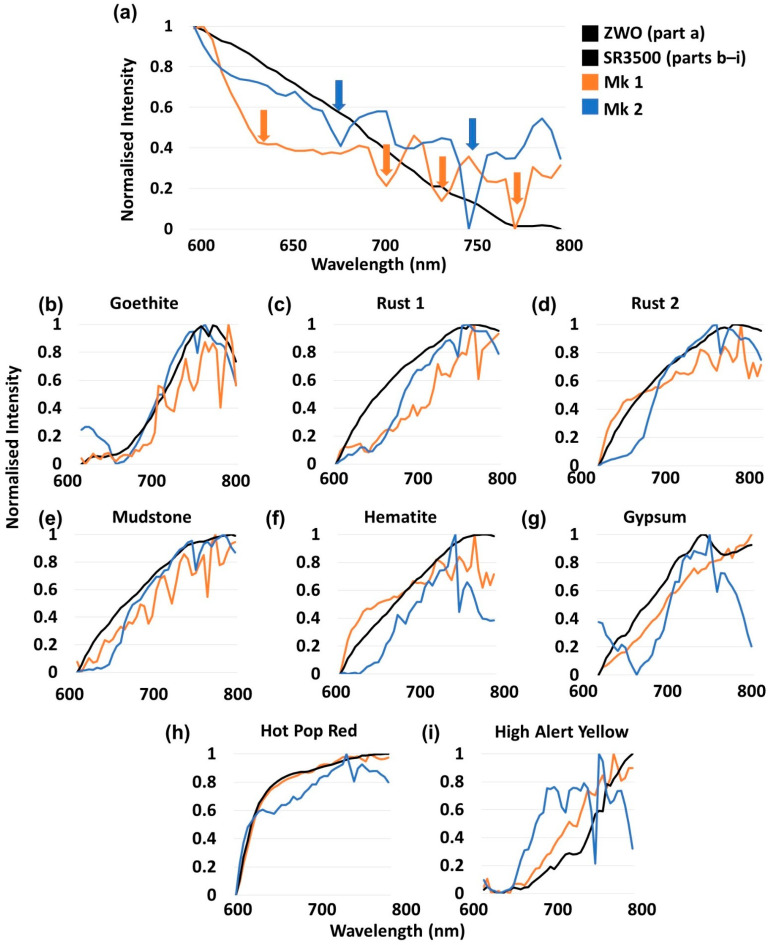
(**a**). RPi Mk 1, Mk 2 and ZWO specular white reflector normalized spectral output. Orange and blue arrows denote absorption regions for the Mk 1 and Mk 2, respectively. (**b**–**i**) Mk 1 (orange) and Mk 2 (blue) normalized intensity curve graphical comparisons with the SR-3500 spectroradiometer (black) for each of the sampled materials.

## Appendix A

Mk 1 and Mk 2 Camera Raw Image Trials.

### Experiment Setup

The experiment with eight of the reflectance materials (Goethite, Rust 1 and 2, Mudstone, Hematite, Gypsum, High Alert Yellow and Hot Pop Red, Figure 1) was repeated for the Rpi Mk 1 and Mk 2 cameras to determine whether capture of raw, unprocessed still imagery would improve correlation with the SR-3500 results. These materials deliberately were selected due a lack of distinctive spectral peaks in their returns and in the case of the iron oxide materials their low reflectance return. Accordingly, full-resolution still images of raw Bayer filter data were captured from these cameras but using full sunlight illumination conditions, as per Section 2.5. The white specular reflectance results for the Mk 1, Mk 2 and ZWO sensor (normalized graphically, Figure A1a) show key differences in the responses of each sensor. The ZWO sensor showed a smooth curve declining in reflectance sensitivity to 800 nm. In contrast, the Mk 1 sensor response dropped sharply at 630 nm and exhibited substantial absorption troughs at 700, 730 and 770 nm (orange arrows, Figure A1a). The Mk 2 camera response exhibited a lower decrease to 800 nm than the Mk 1, and possessed an absorption trough at 680 nm and significant trough at 750 nm (blue arrows, Figure A1a).

Analysis of the reflectance responses from the material subset (Figure A1b–i) revealed a generally improved R^2^ regression of the Mk 1 and Mk 2 cameras (Table A1) compared to the video-derived output (Figure 4 and Figure 5). The Mk 1 camera’s responses exhibited absorption troughs throughout the returns, particularly in the vicinity of 770 nm for all but the Hot Pop Red curves (Figure A1a–g,i). The Mk 2 camera response showed a recurring absorption trough at 750 nm for all sampled materials (Figure A1b–i), concurrent with that identified in the white specular reflection response (Figure A1a, blue arrow).

Sensor responses of the Mk 1 for the iron oxide minerals (Rust 1 and 2, Mudstone and Hematite) revealed a positive increase in reflectance value with an increase in wavelength (Figure A1c–f). The Mk 2 camera response showed an increase for these materials up to 760–770 nm, and then decreased to 800 nm. For the Hematite sample, as well as Gypsum and High Alert Yellow materials, this decrease was pronounced.

**Table A1:** Mk 1, Mk 2 sensor material fitted relationships results.

Material	Mk 1 R^2^	Mk 2 R^2^
Goethite	0.80	0.91
Rust 1	0.80	0.91
Rust 2	0.98	0.91
Mudstone	0.91	0.95
Hematite	0.72	0.69
Gypsum	0.91	0.52
Hot Pop Red	>0.99	0.88
High Alert Yellow	0.85	0.27

The results from the raw Bayer output of the Rpi Mk 1 and Mk 2 cameras demonstrate the effects of the Bayer filter on the spectral curve outputs. Strong absorption troughs throughout both cameras’ output from 600 to 800 nm manifested themselves in the spectral returns. The Mk 1 camera provided more consistent results and presented higher overall R^2^ values than the Mk 2 camera (Figure A1b–i, Table A1). NIR sensitivity response beyond 760 nm for the sampled materials was more consistent than the Mk 2. These factors impacted the performance of these cameras on materials exhibiting low contrast or low overall reflectance in the ferric response spectrum.
